# Challenges and support needs among persons with a migration background who use drugs in four European metropolitan cities

**DOI:** 10.1186/s12954-024-01110-x

**Published:** 2024-11-23

**Authors:** Aline Pouille, Clara De Ruysscher, Lena van Selm, Jan van Amsterdam, Wim van den Brink, Machteld Busz, Roberto Perez Gayo, Marios Atzemis, Wouter Vanderplasschen, the SEMID-EU consortium, Marios Atzemis, Joanna Moura, Ingrid Bakker, Roberto Perez, Sultan Baghdadi, Ralf Köhlein, Astrid Leicht, Mathieu Lovera, Victor Detrez, Elisabeth Avril

**Affiliations:** 1 Department of Special Needs Education, Ghent University, Ghent, Belgium; 2 Barcelona Institute for Global Health (ISGlobal), Barcelona, Spain; 3 Faculty of Medicine and Health Sciences, University of Barcelona, Barcelona, Spain; 4 Department of Psychiatry, Amsterdam University Medical Centers, Amsterdam, the Netherlands; 5 Mainline, Amsterdam, the Netherlands; 6 Correlation—European Harm Reduction Network, Amsterdam, the Netherlands; 7 Athena Hygeia, Athens, Greece

**Keywords:** Migration, Drug dependence, Drug use, Harm reduction, Precariousness, Needs, European Union, Human rights

## Abstract

**Background:**

Persons who migrate for economic reasons, along with asylum seekers and refugees, face multiple personal experiences and societal inequalities that increase the risk of mental health problems and substance dependency, compounded by intersectional social and economic vulnerabilities. The precarious situation and limited access to care of persons with a migration background who use drugs (PMWUD) in Europe raises concern. Therefore, this qualitative study explores the challenges and support needs of a sample of PMWUD in vulnerable situations living in Amsterdam, Athens, Berlin and Paris.

**Methods:**

This study employed a community-based participatory approach. Through semi-structured interviews with PMWUD (n = 99), we identified  (service) needs of a diversity of PMWUD in Europe. Participants were recruited through a combination of community gatekeepers, venue-based sampling, and snowball sampling. Trained community researchers conducted the interviews, which focused on participants’ living situation, substance use, physical and mental health, and employment opportunities.

**Results:**

Despite substantial heterogeneity among the PMWUD, several common themes emerged across all groups. Participants frequently mentioned early childhood adversity, limited social networks leading to loneliness, medical, psychological, and substance use issues, histories of personal violence or poverty, homelessness, lack of necessary documents for health care, social security, and employment, and encounters with the criminal justice system. These intertwined and mutually reinforcing factors simultaneously functioned as barriers to care and support, alongside other barriers such as linguistic and cultural differences, and stigma and discrimination. Due to social exclusion, migration, and substance dependence, participants had limited reliable social networks. Therefore, they often had to rely on accessible and low-threshold services. Harm reduction services played a significant role in providing support to PMWUD. Most PMWUD indicated that basic needs for hygiene and food were met thanks to local organizations. Differences in housing opportunities and access to harm reduction services were identified in each city.

**Conclusion:**

Structural barriers toward treatment and care, often related to administrative requirements, stand in the way of appropriate care for PMWUD. Linguistically and culturally sensitive outreach activities with limited practical requirements could break down social and treatment barriers.

## Introduction

By January 2022, 23.8 million people (5.3%) living in Europe were non-EU citizens. These individuals, who have first-generation migration histories, include both documented (e.g., asylum seekers, refugees, certified labour migrants) and undocumented migrants (living in a country without official residence permits) [1]. The majority of undocumented individuals in the EU initially entered through official channels, holding valid permits for purposes such as studying, working, family reunification, or seeking asylum, but subsequently lost their documented status [2, 3].

International literature on the prevalence of substance use problems among first-generation migrants is limited and inconclusive [4, 5]. The variability in substance use patterns among first-generation migrants is influenced by regional and global differences, contextual factors such as substance availability, and social norms [4]. Typically, first-generation migrants exhibit substance use patterns similar to those of their country of origin, which then evolve to reflect the patterns of their new country over time [6–8]. This ambiguity results from the intricate interplay of various individual, community, and structural protective and risk factors [9]. Protective factors, such as strong social and cultural norms, cohesive ethnic subcultures, and strong religious or familial ties, may safeguard persons with migration histories from substance dependence [4, 10, 11]. Conversely, risk factors linked to pre-, peri-, or post-migration experiences may increase substance use dependence among first-generation migrants. These include social and structural inequities, lower educational attainment, limited job opportunities, and stressors such as acculturation difficulties, poverty, language barriers, mental health issues and trauma [12–16].

First-generation migrants who use drugs, collectively referred to as Persons with a Migration background Who Use Drugs (PMWUD), make up a heterogeneous group. The complex connection between drug use and migration increases homelessness rates among PMWUD. They often live in precarious situations and face multiple health and social problems [17–19]. Municipalities, along with frontline and specialized services such as harm reduction services across the European Union, face the urgent challenge of addressing the high vulnerability of PMWUD [14].

PMWUD may find themselves in precarious and vulnerable situations due to various structural mechanisms and experiences. These include antecedents from their home country, such as war and poverty; their migration journey, such as traumatic migration experiences; and challenges they encounter in the country they reside in after migration, such as discrimination, lack of legal documents, and social challenges [12]. PMWUD are at increased risk of mental health issues due to trauma experienced before, during and after migration, the loss and separation of social networks, and the long-term and accumulated effects of poor health care, chronic stress, and drug dependence [20, 21]. The criminalization of certain forms of homelessness, drug use and migration further exacerbates their situation, leading to stigmatization, marginalization and encounters with the criminal justice system [1, 22, 23]. Such negative encounters disproportionally affect PMWUD, enforcing their vulnerable position in society [23–25].

In Europe, undocumented migrants are openly denied access to social and health rights due to legal restrictions [26–28]. Hence, PMWUD encounter numerous personal, social and societal barriers to fundamental human and social rights as well as to adequate care [29–31]. Research has highlighted an increased risk of HIV and HCV transmission within this population, driven by factors such as poverty, migration-related stressors, social dislocation, forced deportation, infringement on rights, and limited access to healthcare resources [29, 32]. Additionally, overdose deaths and other drug-related risks are more prevalent among those considered ‘vulnerable groups’, such as people experiencing homelessness, many of whom are PMWUD. Harm reduction efforts, by employing outreach strategies, may increase access to care for PMWUD, and thus have the potential to enhance their quality of life and reduce health (care) inequities [30, 31].

PMWUD are considered a ‘hard-to-reach’ population for both health services and research purposes [33, 34]. Services often struggle to reach and adequately support PMWUD, and research focusing on the needs of PMWUD and how services can address these needs in Europe, is scarce [14]. Therefore, the SEMID-EU project^1^ (Services for Vulnerable Migrants Who Use Drugs in the EU), aimed to address knowledge and practice gaps regarding services for PMWUD in the European Union. The qualitative sub-study discussed here was part of the SEMID-EU project and used a community-based participatory research (CBPR) approach to identify the main characteristics and support needs of PMWUD (n = 99) living in four selected European capitals: Amsterdam, Athens, Berlin and Paris. The four cities were selected because they host a high diversity of PMWUD in precarious situations [35]. Paris and Amsterdam have long been known as multicultural melting pots, while Berlin and Athens have seen a significant influx of new migrants in the past decade, some of whom may have migrated for drug-related reasons [13]. Hence, these EU capital cities are confronted with the growing presence of diverse groups of PMWUD facing various health problems and limited access to essential services.

As harm reduction services aim to decrease drug-related risks and harms, they are important services to PMWUD. Hence, PMWUD in vulnerable situations make up a significant share of the service users in harm reduction services in Amsterdam, Athens, Berlin and Paris [13, 36]. Yet, harm reduction services are not always able to reach PMWUD and many questions on the needs of PMWUD remain [33]. This CBPR study aimed to develop an inclusive understanding of the characteristics and needs of a heterogeneous sample of PMWUD in these four cities, with the dual goal of informing both researchers and local harm reduction services. Current challenges in providing adequate support and care for PMWUD in the EU were identified, leading to recommendations for both local services and EU regulations.

## Methods

2.1. Research team and approach.

Community-based participatory research (CBPR) is a community-driven and valid approach for exploring the situations of so-called ‘hard-to-reach populations’ in an ethical and equitable manner [37]. This approach equally involves community members, researchers, and other stakeholders (in this case, harm reduction professionals) acknowledging the unique contributions each brings [38–40]. CBPR aims to create positive and lasting social change by improving health and reducing disparities, through a collaborative process in which all parties strive towards a shared goal [38, 41].

This research was conducted by a team of (1) academic researchers, (2) local practitioners in harm reduction for PMWUD (referred to as local researchers in this study) and (3) community researchers closely connected to local communities of PMWUD. Table 1 provides an overview of the roles of all researchers during the research process. Academic and local researchers were selected for their commitment and expertise and were involved from the project’s inception, including grant writing. Community researchers were recruited by local researchers for their connections with PMWUD, lived or living experiences regarding migration and/or substance use, and willingness and ability to engage in the study. Some participated in early decision-making, while others joined during recruitment and data collection. To increase participation, bimonthly meetings in English were organized with the research team to discuss mutual concerns, questions, progress, and feedback. Community researchers who did not speak English were included in the research process through local researchers, who gathered their feedback and input and communicated it during the meetings. The entire research team also met before the start of the study in Ghent (Belgium) for a two-day, English-spoken meeting with additional experts on the topic and during which the research design was discussed and finalized based on the feedback. The local and community researchers attended a (train-the-trainer) training on recruitment and data collection, which was organised in a common language for the community researchers who did not speak English. All researchers received a compensation for their time invested in this research [40]. The participants received 10 euros in cash or vouchers for participating in the study. The choice of cash or vouchers depended on what the local and community researchers considered best for these participants since there are valid arguments for both choices. The bimonthly meetings continued during the data-analysis phase, ensuring input from all researchers. Several local and community researchers were also involved in the dissemination of the research (i.e., report, scientific articles, presentations and local capacity-building workshops) [40, 42].

**Table 1:** Roles of the academic, local and community researchers

Research activity	Academic researchers	Local researchers	Community researchers
Research design	Developing and writing	Developing and writing	(Developing)*
Ethics application	Obtaining ethical approval	Feedback on ethics application strategies (e.g., informed consent), ethics application	(Feedback on ethics application strategies (e.g., informed consent)), ethics application
Data-collection tools	First and final design	Feedback	(Feedback)
Participant recruitment	Coordination and guidance	Deciding on target communities, developing the recruitment strategy and recruitment of participants	(Deciding on target communities), developing recruitment strategy and recruitment of participants
Data collection		(Conducting interviews)	Conducting interviews
Data management	Data management	Data management	Data management
Data analysis	Analysis of the interviews	Feedback on the analysis, guidance of academic researcher	(Feedback on the analysis, guidance of academic researcher)
Written report	Writing report	Feedback and input for report	(Feedback and input for report)

^*^() Some researchers were involved, but not all

### Participants and recruitment

Three communities of PMWUD were selected for the interviews in each city. These communities were selected because they, according to the local and community researchers, represented significant groups of PMWUD in vulnerable situations with limited access to resources, harm reduction services and care, and little was known about their needs. The twelve selected subgroups of PMWUD and the main migration-, drug- and living situation characteristics of the participants of each community are presented in Table 2.

**Table 2:** Main characteristics of interviewed PMWUD

Community	Age	Gender	Country of origin	Substance use	Legal status	Living situation
Amsterdam (n = 22)						
Intra-European PMWUD (n = 12)	24–53 y/o	11 cisgender male, 1 transgender female	Poland (n = 3), Slovakia (n = 2), Hungary (n = 2), Italy, Lithuania, Austria and Bulgaria (n = 1/country)	Crack cocaine (n = 1) + heroin through injection (n = 11), methadone (n = 7) majority though OAT, cannabis (n = 10)	Mostly European ID’s	Homeless with temporary homeless shelters (in winter)
Arabic-speaking PMWUD belonging to the LGBTQIA + community and involved in chemsex (n = 5)	29–32 y/o	4 cisgender male, 1 transgender	Syria (n = 4) and Lebanon (n = 1)	3-MMC (n = 4, snorting), cocaine (n = 3, snorting) and MDMA (n = 3, pills)	Recognized refugees (residence permit)	Student or social housing
Spanish-speaking PMWUD (n = 5)	33–36 y/o	4 cisgender male	Colombia (n = 2), Peru (n = 1) and Spain (n = 2)	Smoke or snort (crack) cocaine (n = 3), heroin injection (n = 2), + crystal meth (n = 1), + cannabis (n = 2)	European ID’s	Homeless
Athens (n = 20)						
Persons from Maghreb Arabic origin who use drugs (n = 6)	25–40 y/o	6 cisgender male	Morocco (n = 3), Algeria (n = 2) and Tunesia (n = 1)	Cocaine (n = 3) + heroin (n = 2), injectable methamphetamine (‘sisa’) (n = 2), depressants (n = 3)	Residence permit (n = 2), No official residence permits (n = 4)	Homeless
Persons from African origin who use drugs (n = 4)	33–56 y/o	4 cisgender male	Ethiopia, Egypt, Sudan and Congo (n = 1/country)	Heroin (n = 3), (crack) cocaine (n = 2), + cannabis (n = 1), + ’sisa’ (n = 1)	Residence permit (n = 2), No official residence permit (n = 2)	‘Guesthouse’ (n = 3)Homeless (n = 1)
PMWUD residing in the open drug scenes (n = 10)	22–58 y/o	10 cisgender male	Iraq (n = 2), Iran (n = 2), Bangladesh (n = 2), Syria, Pakistan, Afghanistan and Saoudi-Arabia (n = 1/country)	‘sisa’ (n = 10) + heroin (n = 8), + flunitrazepam (n = 4), depressants (n = 4, so-called ‘green pills’)	Asylum status (n = 6) with permanent (n = 2), temporary (n = 2) or unspecified (n = 2) residence permit, No official residence permit (n = 4)	Homeless
Berlin (n = 25)						
Russian-speaking PMWUD (n = 8)	32–50 y/o	5 cisgender male, 3 cisgender female	Latvia (n = 3), Ukraine (n = 2), Moldawia, Lithuania, Belarus (n = 1/country)	Methadone, polamidon or burpenorphine through OAT (n = 7), + heroin (n = 1), + injected polamidon (n = 1), pregabalin (Lyrica) (n = 5), alcohol (n = 4), cocaine (injected) (n = 3) or crack cocaine (smoked, n = 1), and cannabis (n = 3)	European ID’s (n = 3), No residence permit (n = 2), Permanent (n = 1) or temporary (n = 1) residence permit, Registration certificate (n = 1)	Majority homeless, n = 2 used temporary homeless shelters (in winter)
PMWUD from North Africa (n = 10)	21–49 y/o	10 cisgender male	Morocco (n = 5), Algeria (n = 3), and Sudan (n = 2)	(Crack) cocaine, mostly snorted, smoked and sniffed, alcohol (n = 7), cannabis (n = 6)	Majority has no official residence permits (n = 8)	Homeless with temporary homeless shelters (in winter) or relying on friends
PMWUD from West-African origin (n = 7)	21–48 y/o	7 cisgender male	Republic of Guinea (n = 2), Sierra Leone, Niger, Mauritania, Ivory Coast, Angola (n = 1/country)	(Crack) cocaine (mostly smoked) (n = 6), cannabis (n = 5), alcohol (n = 3)	Majority has no official residence permits (n = 5)	Homeless with temporary homeless shelters (in winter)
Paris (n = 32)						
PMWUD from Georgian origin (n = 16)	25–62 y/o	15 cisgender male, 1 cisgender female	Georgia	Subutex® or buprenorphine, methadone through OAT (n = 15), + heroin (n = 4), + injecting methadone (n = 2), cocaine (n = 9), prescribed depressants (n = 7), alcohol (n = 9)	Majority has no official residence permits (n = 14), 2 had an asylum status	Majority lives in housing projects, 3 are homeless
Non-Georgian Russian-speaking PMWUD (n = 10)	25–47 y/o	6 cisgender male, 4 cisgender female	Lithuania (n = 3), Latvia (n = 2), Chechenia (n = 2), Belarus, Ukraine, Moldavia (n = 1/country)	Methadone (n = 9) the majority through OAT, + heroin (n = 2), + morphine (n = 1), (prescribed) depressants (n = 6)	No official residence permits (n = 5), European ID’s (n = 5)	Majority lives in (temporary) housing programs, social housing or with friends, 1 is homeless
Persons from Somalian origin who use drugs (n = 6)	25–35 y/o	6 cisgender male	Somalia	Crack cocaine (n = 4), cannabis (n = 4), ‘tablets’ (n = 2), depressants (n = 2) methadone (n = 1), alcohol (n = 1)	Majority has temporary residence documents (n = 4)	Homeless with temporary shelter (in winter)

Local and community researchers recruited participants using purposive sampling [43] through three different routes:

- Via gatekeepers: mediators who introduced people they thought were eligible for the interview and asked them to participate or suggested that they contact the researchers [44].
- Venue-based recruitment: researchers visited places regularly frequented by PMWUD to reach and recruit participants [45];
- Snowball sampling: participants were asked whether they knew others who met the inclusion criteria, and if so, to inform them about the research and possibilities to participate [46].

While the community researchers were able to overcome many barriers in getting access to PMWUD, recruitment proved to be challenging in some communities. This depended on community, city (services) and researcher characteristics. Among Spanish-speaking PMWUD in Amsterdam, for example, stigma surrounding drug use, and related inaccessibility of services for Spanish-speaking PMWUD, challenged the recruitment of Spanish-speaking PMWUD in Amsterdam. In Athens, next to service-related barriers toward reaching PMWUD, the community researcher did not have a migration background, increasing language and other barriers toward migrant communities. While these barriers were partly overcome by ongoing recruitment through persons with similar lived experiences in venues where PMWUD were present (e.g., harm reduction services, local community services, in open drug scenes or the streets), this sometimes resulted in unequal representation of communities within the cities.

### Data collection and analysis

Following pilot interviews conducted in German, English, Georgian and Arabic, the final semi-structured interviews (which took approximately 30 to 40 min) aimed to collect information about: a. migration background and status; b. living situation (i.e., daily occupation, social network, basic needs); c. patterns of substance use; d. physical and mental health problems; e. specific support and service needs; and f. experiences with criminal justice and law enforcement. The interview protocol, developed in collaboration with the entire research team of academic, local and community researchers, was available in English, Arabic, French, Georgian, Polish, Russian, Somali and Spanish.

The interviews were audio-recorded, transcribed verbatim and translated into English by a professional translation service. The academic researchers familiarized themselves with the data and discussed any questions with the local and community researchers. To analyze the data, we employed a directed qualitative content analysis approach [47]. In the first phase, the academic researchers analyzed the interview data separately for each city, organizing the findings by community and categorizing them according to predefined themes derived from the questionnaire. This allowed us to identify key patterns and insights specific to each community within the cities. The academic researchers then conducted a comparative analysis across the different communities within each city, identifying commonalities and differences. This process resulted in a comprehensive report of the findings for each city, which was reviewed and refined with feedback from academic, local, and community researchers. Finally, the academic researchers carried out a cross-city comparison by aligning the results under each theme across the four cities. This multi-level comparative analysis enabled to identify both broader trends and more localized variations, providing a nuanced understanding of the similarities and differences in the experiences of PMWUD in diverse communities and urban settings. While the full report [see 48] provides an in-depth look into the situation of PMWUD in the four cities, including insights from additional multidisciplinary focus groups, this paper primarily focuses on challenges faced by PMWUD across the four cities and their implications for policy-making in Europe.

## Results

This section provides a general overview of the main characteristics of the participants, followed by overarching findings that emerged across the communities in all cities. Finally, specific results for each city are presented in detail.

### Study participants

**Fig. 1:**
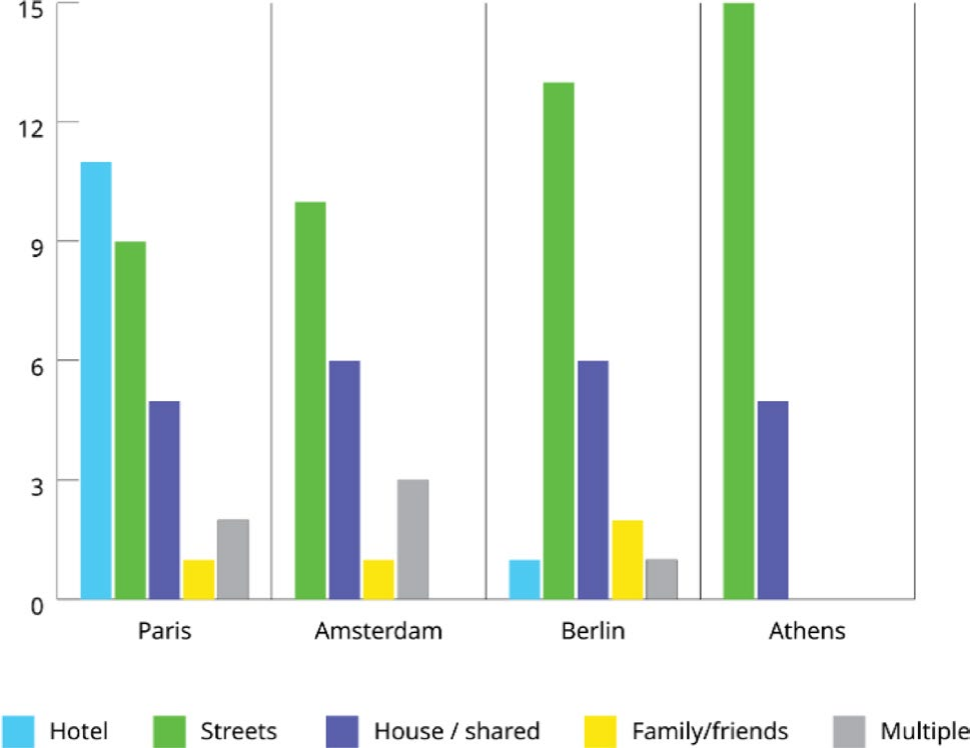
Living situation of participants

Across the four cities, in total 99 participants from 43 different countries of origin and 45 different nationalities were interviewed in 14 different languages. The vast majority of participants identified as cisgender men. Eight cisgender women (all Russian-speaking) and two transgender women participated in the interviews. In all cities, except Paris, where many relied on a housing program, most of the participants indicated that they were experiencing homelessness (see Fig. 1). Additionally, most participants in Paris, Berlin and Athens lacked official residence papers (see Table 2).

Across the cities, participants exhibited diverse substance use patterns, including oral and intravenous consumption. However, within ethnic communities, substance use patterns often showed similarities. Commonly reported substances included stimulants (primarily cocaine) and opioids (predominantly heroin and methadone), alongside depressants (mostly alcohol), cannabinoids, and, notably among the community of LGBTQIA + engaged in chemsex, dissociatives, empathogens and psychedelics.

### Overarching findings

The diversity among the population of PMWUD implies the complex and highly individualized nature of their support needs. Despite this heterogeneity, we identified several overarching findings.

PMWUD without legal documents such as an ID, residence permit or health insurance, expressed a high need for administrative support to obtain or renew these essential documents. The absence of official identification, residence or work permit, and health insurance was cited by nearly all migrants as a major obstacle hindering access to housing, social security, medical care and recovery on multiple life domains. The stress and uncertainty associated with the lack of official documents were identified as direct contributors to increased substance use, involvement in criminal activities and heightened psychological distress, perpetuating a cycle of vulnerability that is difficult to escape. Outreach initiatives played a pivotal role in delivering support and overcoming geographical, practical and stigma-related barriers. In Athens, for example, so-called ‘street lawyers’ proved instrumental in assisting PMWUD by addressing legal barriers and facilitating access to necessary documentation and services. Having an asylum or refugee status generally improved access to care and resources, but temporary residence permits often led to instability in the lives of those impacted. There were clear disparities in access to resources based on country of origin and documentation status. For instance, PMWUD from Ukraine appeared to receive better support than others, impacting their well-being and opportunities for personal and social development. This was likely related to the Russian invasion of Ukraine and welcoming EU policy towards Ukrainian refugees.

Many participants faced homelessness, as stable housing often required a residence permit. Notable housing programs for undocumented PMWUD were identified in Berlin and Paris, such as the Assore program in Paris, which reduced street homelessness by providing hotel accommodations. In Amsterdam, many participants from Maghrebi countries had stable housing, while many PMWUD in Athens were homeless, relying on temporary and inadequate shelters, contributing to feelings of instability, uncertainty and unsafety. Across all cities, lacking a home address posed a barrier to obtaining essential legal documents, health insurance, and work permits, as these often require a fixed address or contact point.

Social networks were limited due to factors such as migration, homelessness, substance use, stigma, and associated feelings of distrust, making it challenging for PMWUD to rely on family or a community for support. Consequently, they often turned to professional services for personal needs and, to a lesser extent, social support.

Basic needs such as hygiene and food were generally met in all four cities through services providing showers and meals. However, access to healthcare remained a significant issue due to the lack of legal documents. Many PMWUD had unmet medical needs related to substance use and homelessness but could rely on harm reduction services for urgent care. Screening and treatment for infectious diseases were generally available, especially in Amsterdam and Paris.

Opioid agonist therapy (OAT) was accessible to persons with temporary or EU documents in all cities except Athens, helping participants reduce harmful substance use and gain stability in their lives. Among those without legal residence, however, OAT was generally not accessible, except when the harm reduction services were able to overcome legal barriers through creative solutions. In Paris, for example, OAT was sometimes offered anonymously (although including a medical screening and pseudonymized patient file), which increased access for undocumented PMWUD. Waiting lists in Amsterdam posed a challenge to PMWUD. Participants emphasized the need for culturally sensitive and linguistically accessible information on drug consumption, harm reduction, drug treatment and support in multiple life domains. This was exemplified in Paris, where Russian-speaking personnel within the DCR increased access for Russian-speaking PMWUD.

The need for mental health support among PMWUD varied significantly based on their individual circumstances and the root causes of their mental health issues. Participants often prioritized addressing their precarious living conditions when identifying their needs, especially when those conditions were perceived as significant contributors to their mental health challenges. In contrast, individuals with relatively stable living situations who experienced severe trauma, often linked to their migration trajectory, expressed a clear need for mental health support. However, accessing this support proved challenging due to various barriers, including legal issues (such as lack of documents), language differences, cultural factors, financial constraints, and lack of awareness about available services. Substance use frequently emerged as a coping mechanism to manage both mental and physical hardships among PMWUD. This role of substance use as a dual coping mechanism –with mental health issues and with physical challenges – underscores the complex interplay between mental health, substance use, and broader social determinants affecting this population.

Employment was a major challenge among the vast majority of the PMWUD due to barriers such as homelessness, substance dependence, and lack of a work permit. Participants highlighted a vicious cycle wherein unemployment, undocumented status, homelessness, and substance use reinforced each other, underscoring the urgent need to reduce these barriers to employment.

The majority of PMWUD had interactions with the criminal justice system in these cities, involving encounters with police and the courts. These interactions were often linked to interconnected issues, such as homelessness, substance use, financial hardship, migration, undocumented status and stigma. Participants reported diverse experiences with law enforcement and detention facilities. Some described fear-inducing, harmful and stigmatizing encounters with the police, leading to increased vulnerability, including, the accumulation of fines. However, others recounted instances where police referrals to shelters and harm reduction services were helpful.

Involvement with the criminal justice system could have a detrimental impact on the documentation status of PMWUD. Conditions in prison were often perceived as preferable compared to living on the streets, sometimes (depending on documentation status and city) offering OAT and/or screening and treatment of communicable diseases such as HIV, hepatitis and tuberculosis. After release, however, a lack of continuity in support was observed and PMWUD frequently faced accrued debts of utility costs in prison.

### Amsterdam

#### Characteristics of the sample

In Amsterdam, interviews were conducted with a diverse group of 22 PMWUD. This group includes twelve intra-European labour migrants, five Arabic-speaking PMWUD identifying as LGBTQIA + and involved in the chemsex community, and five Spanish-speaking PMWUD.

The intra-European participants, aged 24–53 years and predominantly from Eastern Europe, had migrated to Amsterdam between 5 months and 10 years ago. The LGBTQIA + participants, aged 29–32 years, had migrated 6–8 years ago, seeking refuge from war or persecution based on their sexual orientation in their home countries. This group generally faced less precarious situations concerning drug dependence, housing stability and encounters with the criminal justice system compared to the other communities. The Spanish-speaking participants, aged 33–47 years, had migrated between two weeks and three years before the interviews. Most participants either possessed European identification documents or, among the Arabic-speaking PMWUD, held a recognized refugee status.

#### Substance use

Among the intra-European PMWUD, daily use of (crack) cocaine and heroin was common, primarily administered through injections. Some also mentioned combining heroin and cocaine. Ten participants used cannabis regularly, and seven used methadone daily, mostly through Opioid Agonist Therapy (OAT). The main reason for using drugs was physical dependence and to deal with homelessness, depressive feelings, feeling unsafe or uncomfortable, and to feel “at peace”, and “forget problems”. The Arabic-speaking participants identifying as LGBTQIA + knew little about drugs and their risks, used substances such as 3-MMC, cocaine, and MDMA at social events and during sexual activities. Driven by loneliness and stress, they encountered drugs rather easily through their social networks. Other reasons for drug use included boredom, trying to ‘escape’ from reality and trauma experienced in the past. Spanish-speaking participants did not inject drugs but frequently smoked or snorted (crack) cocaine, sometimes in combination with heroin or crystal meth. They all used drugs to survive on the streets: socially (using with peers), to deal with boredom and to escape from the harsh life and lack of perspective. Spanish PMWUD were rather reluctant to open up about their substance use problems, presumably because of the stigma surrounding drug use.

#### Social network

In terms of social networks, intra-European PMWUD typically had limited social connections and were unfamiliar with the local healthcare system, often due to language barriers. The Arabic-speaking PMWUD, although facing challenges integrating into Dutch society, had some social support networks but often experienced loneliness. Spanish-speaking participants also described feelings of loneliness and distrust, with limited social networks partly due to shame surrounding their circumstances.

*“Here, I’ve personally noticed a level of, well, a point where my mind starts to slip due to desperation, and especially due to loneliness. It’s worse than work and everything else because extreme loneliness directly destroys your ability to communicate and do many things. That’s where you start becoming a person who can be frightening, who can become extreme due to powerlessness. If you’re psychologically unwell and you need medication but can’t afford it, you’re screwed.”* (R., male, 36 yrs., Spanish origin)

As R. described, loneliness and related desperation could be a catalyst for further marginalisation and substance use. As V. further indicates, loneliness is linked to migration and the resulting lack of sources of support.

*“The feeling of loneliness is very scary. Unfortunately, one comes to this country alone, he has to deal with his problems by himself, so he’s going to struggle alone, he’s going to do everything alone. It’s a lot for one person to deal with, and it definitely has its toll on one’s health and psyche*.” (V., male, 31 yrs., Lebanese origin)

#### Medical and mental health problems

Participants across groups reported various medical and mental health challenges, including psychotic episodes, paranoia, depression, and stress related to their living conditions and drug use. LGBTQIA + participants expressed the need for mental health support, especially for PTSD.

*“The support I currently need is someone to listen ... because when I was undergoing trauma treatment, someone was there for me... and now no one is there for me. (…) When I’m not in a good mood, drugs are my only escape, so I do them.”* (S., transgender woman, 29 yrs., Syrian origin)

Due to the snorting of drugs, Spanish-speaking participants stated to have dental problems.

#### (Support) needs

In contrast to the Arabic-speaking participants who lived in (student) houses, the Spanish-speaking PMWUD referred to housing as a “top priority” to escape from the harsh living conditions on the street. Likewise, ten intra-European participants indicated the need for long-term and stable housing conditions where they could feel safe, in contrast to the everyday struggle and uncertainty of homelessness.

Across communities, many participants indicated to have depressive and stressed feelings. The PMWUD expressed the need for more connectedness and belonging, financial resources and psychological support for trauma (LGBTQIA + participants). On entry into Amsterdam, LGBTQIA + participants experienced a lack of support related to migration procedures (e.g., asylum request, legislation and customs), mental health, and drug use and services.

Despite receiving state benefits such as housing and student grants, most LGBTQIA + participants indicated that this support was insufficient.

*“That [support with employment] is what we need. After all, I didn’t come here to be a homeless person, I didn’t come here to become a junkie, waste my life, or end up in jail. We came here to work, to change our lives, to have a better lifestyle.”* (R., male, 36 yrs., Spanish origin)

Across all groups, having a job was mentioned as an important resource, both financially and as a distraction from substance use.

#### Harm reduction and other types of support

In contrast to the LGBTQIA + participants, all intra-European and Spanish PMWUD were in contact with at least one support service related to their basic needs and drug use. These services helped them with food, hygiene and shelter, and assisted in arranging social affairs (e.g., short-term housing, help with paperwork, fines) and medical needs. This support was provided even if they did not have access to insurance, including OAT, dental care and other health care services. One Spanish-speaking person and all intra-European participants had been tested for hepatitis, tuberculosis or HIV and were treated if positive. Among Spanish-speaking participants, drug consumption rooms (DCRs) were not often used. Those who did indicated that it provided them with a judgement-free place connecting them with professional help and enabling the prevention of drug-related medical complications.

*“Fortunately, there is this place that, at least you feel kind of at home, safe. That’s important and warm. Yes, and also you don’t feel so illegal. Cause when you are on the street, um, you feel that you do something that not everybody likes. So, I appreciate a place like [harm reduction service] that is open here, and let us use, you know, in a healthy and quiet way.”* (F., male, 42 yrs, Italian origin)

Hence, DCRs were not only helpful for health-related reasons but could also positively impact participants’ feeling of belonging to a stigma-free environment.

#### Barriers to care and other types of support

While some participants received support from local services, many faced barriers related to language differences, lack of awareness about available services, and stigma associated with drug use. People were on a waiting list, because of the limited dispensing of methadone to people without insurance. To increase the accessibility of support services, Arabic-speaking PMWUD mentioned the importance of Arabic or English-speaking therapists to reduce waiting times and offer culturally and linguistically tailored trauma treatment.

*“A professional mental health therapist should be assigned the task of treating people who come to the country. I mean, when I arrived in this country with all the “traumas” (…) I do not see a professional psychologist, because I’m a refugee and I don’t speak the language, so I have to be on a long waiting list to see a psychologist who speaks English, not Dutch. So, this is definitely something we need.”* (V., male, 31 yrs., Lebanese origin)

PMWUD expressed a significant need for information on drugs and drug services in different languages and relevant contexts. Physical dependence and withdrawal symptoms, linked to a lack of access to methadone, formed barriers to work and a citizen service number. The latter can only be obtained when having a home address. One participant highlighted the challenge of the first month without income, where meeting basic needs, (such as accessing food distributed at specific times) becomes a daily struggle and stands in the way of focusing on the job. Several Spanish-speaking PMWUD mentioned language barriers, homelessness, substance use, and restrictions to using OAT-medication at work as barriers to employment. Eligible people face waiting times of up to ten years for social housing resulting in people being placed on waiting lists for shelter, especially in summer. Other challenges included living among other persons who use drugs in shared shelters and negative experiences with services related to not feeling heard.

#### Encounters with the criminal justice system and law enforcement

N., explained how his migration history, related poverty and lack of social network led him to engage in criminal activities.

*“I go Friday, to go and steel. And then, on Monday, I go in new clothes in school. I have not papa. I have not mama but I have this and I was motivated this way.”* (N., male, 24 yrs., Slovak origin)

Hence, almost all intra-European migrants had spent some time in prison, either in the Netherlands or abroad. Though the conditions in prison were generally described as comfortable compared to conditions on the outside, methadone was not sufficiently provided, posing potential health risks. The Dutch police were mostly described as friendly, social and empathic, especially when compared to police forces in some countries of origin such as Austria and Poland). Four participants even received help from the police in finding shelter and accessing OAT. Nevertheless, some received fines for precariousness-related acts such as sleeping on the street or not paying for public transport.

*“I got a fine for sleeping outside, for being homeless. This is really crazy, huh? (…) And then, you get a fine for sleeping outside, and then you cannot pay, and they need to put you two days in prison. (…) If you tell me in this country, it’s not possible to be homeless, instead of getting a fine, they need to get your place to sleep.”* (R., male, 36 yrs., Austrian origin)

Since they could not pay these fines, this could lead to a build-up of debts and confinement.

### Athens

#### Characteristics of the sample

In Athens, 20 PMWUD were interviewed: six persons of Maghrebi origin, four of Sub-Saharan African origin and ten residing in the open drug scenes of Athens, from various countries of origin. Participants from Maghrebi countries in North Africa (aged 25–40 yrs.) migrated 2–16 years ago and most of them spoke Greek. Participants from the sub-Saharan African community (aged 33–56 yrs.) migrated to be reunited with their families or to seek political asylum. Participants from the open drug scenes (aged 22–58 yrs.), came 5–34 years ago from various countries and were mostly war refugees or were prosecuted in their home countries. Two Maghrebi PMWUD and two from sub-Saharan Africa had a valid residence permit. Six migrants from the open drug scenes had an asylum status and therefore a residence permit.

#### Substance use

Participants of Maghrebi origin exhibited varied drug use patterns: three used cocaine daily (two also used injected heroin), two smoked adulterated methamphetamine called ‘sisa,’ and three used depressants or flunitrazepam. None received OAT. Their drug use occurred mainly on the streets, driven by physical dependence, the need for “courage”, or to forget problems from precarious living conditions and loneliness. Participants from the open drug scenes often used sisa (n = 8), heroin (n = 8), and/or flunitrazepam (n = 4), mostly on the streets (n = 8) and/or at DCRs (n = 5). None of them received OAT. One used sisa to quit drinking and heroin, while others used drugs out of boredom and to cope with loneliness.

One sub-Saharan PMWUD on OAT used cannabis daily. Others used heroin (n = 3), crack cocaine (n = 2), cannabis (n = 1), and sisa (n = 1), mostly on the streets. They described the intertwinement of homelessness, undocumented status, unemployment, sadness and loneliness, drug dependence and encounters with criminal justice.

*“I was in a situation as I am now, unemployed, I had no job, I had nothing. I got busy using drugs. Using and using, then I said if I use, where am I going to get money? (…) 13 grams kept me in prison for 12 years. They had me on the internal market and as a dealer. But for that to tear up my papers? So, I wouldn’t go home to see my mother [crying]. I haven’t seen her since.”* (G., male, 44 years old, Bangladeshi origin)

#### Social network

Most PMWUD lost contact with their family, which often led to emotional struggles which they attempted to numb through substance use.

*“I have missed them very much. And it gets me down in the dumps. Especially when I see a family with a father, a mother, and small children. I speak to myself and say: ‘Where are mine?’”* (J., male, 34 yrs., Syrian origin)

Most did not have any friends to rely on, except for five participants who mentioned a small network of people with similar migration backgrounds who also used drugs on the streets, providing them with some sort of support. Others mainly described how the harsh living conditions on the streets lead to feelings of mistrust and social isolation.

#### Medical and mental health problems

Across all communities, PWMUD faced significant mental health problems: several experienced hallucinations, depressive feelings, sadness, depression, and suicidal thoughts. Mental health issues ranged from schizophrenia to general psychological distress. Physical health concerns were also prevalent, including infections such as hepatitis C and HIV, and conditions such as diabetes and heart problems. These health issues were compounded by the harsh and unsafe conditions of street living, which contributed to fear, insomnia, and mental health problems. Depending on the source of these mental health problems, some expressed a desire for psychological support and substance use treatment.

#### (Support) needs

The most pressing needs were stable housing, including amenities such as electricity and warm water, and residence permits. Most PMWUD of Maghrebi origin and those living in the open drug scenes were homeless, occasionally finding shelter in guesthouses or emergency shelters. In contrast, sub-Saharan PMWUD generally had more stable living situations, with three residing in a guesthouse. Organizations such as emergency night shelters played a crucial role in meeting the basic needs of food and hygiene for the majority of participants. Some PMWUD received legal support from so-called ‘street lawyers’ (i.e., outreach lawyers from social welfare and harm reduction services) to obtain permits. Due to unemployment, seven persons needed financial support. PMWUD mentioned the need for residence papers to visit family and friends in their countries of origin and reported feeling lonely and guilty about not being able to do so. Homelessness was clearly linked to feelings of fear, loneliness, involvement in criminal activities, and the loss or lack of residential papers, exacerbating substance use. Hence, finding a stable home was highlighted as a critical support need to address these challenges.

*“Basically, you live under constant fear that you will be robbed, or they will beat you up causing you to be unable to get any sleep, which in turn brought me insomnia. (…) I cannot take it anymore, I do not know how to cope with it, it’s hard to live in the streets and not to have a house, a place to stay in if you understand me. (…) First things first, I would want to rest properly and have a place to stay, and afterwards, I want to continue searching for a job because I’ve been already searching for one but in my current condition, it hasn’t been going well. I’m tired of not having my own shower, electricity, a roof above my head and my base utilities. (…) If I had those I would stay away from drugs.”* (Y., male, 38 yrs., Tunisian origin)

#### Harm reduction and other types of support

Some PMWUD pointed to harm reduction services and drug treatment centres as helpful for providing amenities such as a shower, as well as psychological and medical support. Participants also mentioned the value of an emergency shelter providing food and street lawyers providing legal support to homeless people for obtaining residence and other permits.

*“That’s why I used, because I was without a house, without a card, I can’t work. Mrs H., the lawyer, helped to give me an asylum card. I wanna work in dishwashing. Washing dishes, something else to pass the time, the day. To make it in time to sleep at [night shelter].” *(S., male, 25 yrs., Iranian background)

While psychological support was not frequently mentioned as a priority need, three sub-Saharan PMWUD and two from the open drug scenes mentioned that having access to a psychologist to talk to would be helpful.

#### Barriers to care and other types of support

Participants indicated that negative past experiences of “not trusting”, “not liking” services, or “not feeling heard” prevented them from being in contact with (harm reduction) services. Additionally, three participants could not locate the services, and others avoided returning due to feelings of shame about failing to quit substance use or perceived favouritism towards Greek natives in the shelter provision. Stigma related to substance use and the lack of legal documents created barriers to job opportunities. This sense of hopelessness, compounded with homelessness, often led to substance use as a coping mechanism.

*“On the street. I had no home. On the street, nobody helps me from Iran and why be burdened with me? Sisa helps you pass the time.”* (S., male, 25 yrs., Iranian origin)

#### Encounters with the criminal justice system and law enforcement

Half of the Maghrebi PMWUD, five participants from the open drug scenes and one sub-Saharan PMWUD had been incarcerated in the past, mainly for drug- and precariousness-related offences. Participants who had spent time in prison linked prison to drug use but were also positive about being tested for HIV and hepatitis C, as well as receiving treatment for diagnosed illnesses. Two PMWUD were unable to pay the fines and bills for expenses accumulated during their time in prison, leading to fear of being arrested by the police. This fear was also prominent among two others who lacked legal residence papers. Due to their substance use, one Iranian participant discussed that he was severely physically abused by the police, while another expressed fear of discrimination by police officers. Nevertheless, the majority of the participants had no problems with the police. Police officers were even described positively, and one participant talked about receiving advice from police on how to handle street violence.

### Berlin

#### Characteristics of the sample

In Berlin, 25 PMWUD were interviewed: eight Russian-speaking, ten from North African countries, and six from West Africa. The eight Russian-speaking participants (32–50 years old) migrated 1–13 years ago and had varying citizenship statuses affecting their residence permits. Three with Latvian/Lithuanian nationality had European IDs, though one lost theirs. Two had no official ID, one Ukrainian had a permanent, another a temporary residence permit, and one from Belarus had a registration certificate. Three, including two with EU passports, lacked medical insurance. The North African participants (21–49 years old) fled from their home country 1–14 years ago and faced challenges related to asylum status, housing, jobs, family, language, healthcare, integration, and discrimination, affecting their mental health. Many lacked certified documents, making employment illegal and unsustainable. Six requested asylum, but only two had temporary residence permits. Others had no official ID due to expired or unreceived permits. The seven West African participants (21–48 years old) migrated 5–15 years ago. Three fled wars in Sierra Leone, Mauritania, and the Ivory Coast, with two holding temporary residence permits. Others migrated for family issues or to study and lacked official ID or needed permit renewals. Many did not wish to stay in Germany but could not return home due to financial and ID constraints. All were homeless, staying with friends or in shelters without fixed residences.

#### Substance use

All but one Russian-speaking participant were receiving OAT with methadone, polamidon, or buprenorphine. This could be related to increased accessibility due to the presence of a Russian-speaking counsellor in the harm reduction service. In addition to OAT, Russian-speaking participants commonly used pregabalin (Lyrica) (n = 5), alcohol (n = 4), injected cocaine (n = 3), smoked crack cocaine (n = 1), and cannabis (n = 3). Participants often used drugs in public places such as streets, parks, public toilets, or subway stations. Three participants used DCRs when accessible, depending on the location and opening hours. Most of the Russian-speaking participants began using illicit substances in their home countries between the ages of 14 and 29. In contrast, none of the sixteen West or North African PMWUD used opioids. The main problem substance in these groups was (crack) cocaine (n = 12 on a daily basis). North African PMWUD snorted (n = 3), sniffed (n = 2) and smoked (n = 3) substances, whereas all but one West African participant smoked (crack) cocaine and twelve of them also used cannabis, seven of whom used it daily. Most North African and three West-African participants used alcohol. The twelve West African and the North African PMWUD for whom data on initial substance use was available, started drug use in Germany and used in public spaces.

Substance use was generally driven by the need to cope with psychological issues stemming from migration-related stress, such as the uncertainty related to obtaining official documents and permits, deportation, loneliness, trauma, grief, and unstable living conditions. Boredom from street living and unemployment also played a role.

*“When you can’t find peace of mind and you’re depressed, of course you’re going to do drugs. When you sleep on the street, you only have to worry about yourself. (…) The financial situation and the psychological condition... It’s the snowball effect. (…) I know that drug abuse is not good, but because of stress, the psychological factor, sleeping on the street, one finds himself in a situation where he has to do it. What am I supposed to do? There is no other solution.”* (B., male, 47 yrs., Sudanese origin)

Many PMWUD reported that their substance use evolved from an urge to get high as a means of coping with these challenging circumstances.

#### Social networks

Most participants’ social networks consisted of persons with similar migration backgrounds who also used drugs and lived in public spaces, whom they regarded as friends. However, friendships with these peers were often considered difficult because (a) their precarious living situations led to priorities other than maintaining friendships, or (b) their substance use was triggering for those trying to (re)gain control over their substance use. Two Russian-speaking participants felt alone, partly because they could not communicate with German-speaking peers. Eight North African participants also suffered from loneliness, isolation and feelings of “merely depending on themselves”, which, as indicated by two participants, contributed to their psychological stress and substance use. The large majority of PMWUD had no family or had lost connections with their families due to substance use and/or migration, which was often a cause of emotional pain and distress.

*“You see, my son is 16 years old now, you understand what period of life it is? It’s the most important one. (…) And nobody is there to watch over him. (…) You touched on this topic and I already have tears.”* (S., male, 50 yrs., Ukrainian origin)

#### Medical and mental health problems

The Russian-speaking PMWUD had many medical needs, primarily dental problems. Six had been tested for HIV, tuberculosis and hepatitis, with five testing positive for hepatitis C (three were treated). One participant tested positive for tuberculosis (treated once incarcerated). Half of the Russian-speaking participants tested positive for HIV. While Ukrainian PMWUD received full support for HIV treatment, antiretroviral therapy and OAT, migrants from Latvia, Moldova and Belarus could access these resources only through special compensation systems.

*“I was taking strong painkillers after my car accident. I have bad injuries. I was taking opium-based painkillers. Since I had no insurance in Germany, no access to medication, I started to take heroin as a substitution for painkillers.”* (A., male, 37 yrs., Latvian origin)

A.’s story showed how a lack of insurance can have a devastating and long-lasting effect on PMWUDs health. The other participants reported fewer testing of communicable diseases and medical needs. Participants across all three communities (though less among the West African participants and most prevalent among participants from North Africa) reported mental health problems related to the stress of their living situation and visa insecurity.

*“You find peace of mind when you have a place and ID documents like everyone else. When you do not have ID documents, a place, and have a drug addiction, how could you find peace of mind? As I’m sitting with you right now, I can’t find peace of mind because I don’t have a place, I don’t have a wife, I don’t have children, I don’t have ID documents, I don’t have-- I don’t even have an income source, frankly... How could I find peace of mind? Psychologically, I feel distressed.”* (S., male, 49 yrs., Moroccan origin)

PMWUD further described their mental state as “depressed”, “sad”, “mentally and emotionally broken” and “living in darkness”. Few participants said that traumatizing experiences from the past dominated their mental health.

#### (Support) needs

Housing stability was a major concern among the PMWUD. Only two participants of North African origin had stable housing. Six West African participants were partially homeless, staying with friends, while others lived on the streets but sometimes found winter shelter in churches or emergency shelters. Four Russian-speaking participants mostly lived on the streets, occasionally using a homeless shelter despite safety and hygiene concerns. The other Russian-speaking participants lived in community housing with access to basic needs such as showers and electricity, although privacy was a concern. Two West African participants indicated access to basic needs as one of their most prominent support needs. Most participants on OAT found it helpful for regaining control over their lives. Five Russian-speaking migrants highlighted the importance of OAT and care for dental, gastrointestinal, or heart issues, which they received through insurance or support services. Navigating the German bureaucracy was challenging for many, partly due to language barriers.

*“In Berlin, or Germany in general, there is an extreme amount of bureaucracy. They acknowledge it themselves. When they need “this paper and that paper” then, to get them, you would have to go back and forth, from one place to another. If you don’t know the German language well, then it’s going to be your worst nightmare. It’s so difficult for me because the head office is 80 kilometres away from where I live. It’s not here. I go there every couple of months to extend [the temporary residence permit].”* (S., male, 50 yrs., Ukrainian origin)

After basic and medical needs were fulfilled, participants started to express the need for employment or other ways to spend their day purposefully (e.g., education, doing sports). Except for two North African participants with official residence permits who received 300–410 EUR/month, financial support was often mentioned as a need.

#### Harm reduction and social services

The Russian-speaking PMWUD mentioned harm reduction services, general practitioners and community shelters as important resources of help because they provided OAT, social support, and assistance with housing, paperwork and access to medical provisions.

*“They [PMWUD] should receive substitution therapy. There’s an opportunity to receive it even without insurance, like in my case. Then all sorts of things are provided by the social workers. If everything is fine with your papers, you can get on Mini Job. You will have a beautiful and happy life.”* (M., male, 42 yrs., Lithuanian origin)

As M. discussed, offering PMWUD opportunities to receive support for drug problems, legal issues and employment, may have a long-lasting effect on their well-being and society at large.

In particular, one harm reduction service was mentioned as helpful because of its late opening hours and Russian-speaking counsellors that decreased language and cultural barriers. Some described how these counsellors became role models to them over time, as they could identify with them and admired them. Three participants underscored the importance of syringe distribution services, DCRs and a mobile van to use substances in a safe, non-public space. Two Russian-speaking PMWUD expressed the need for more recovery-oriented support that would enable them to find a job and be connected to people who do not use drugs*.* Seven West African participants were in contact with harm reduction services for the provision of safe drug consumption materials, emotional and practical support, clothes and food. Six of them received no support, saying they needed to “deal with their problems on their own”, referring to problems with paperwork, psychological problems, food, drugs and financial challenges.

#### Barriers to care and other types of support

Not having official residence papers and IDs, together with the related risk of being deported raised fear to contact services, especially when the police controlled the public spaces where harm reduction services operate. Across all three communities, the most important barrier to care was not knowing where to find it and the lack of legal documents required for access to services. Language barriers not only exacerbated these access issues but also hindered mental health consultations and medical care due to mutual misunderstandings. Multilingual services were advocated to overcome these barriers.

*“So, if a person doesn’t know the language, he doesn’t have access to the so-much-needed information. I met many people who don’t even know that there is such a thing as Berliner Stadtmission, a place where one can stay for the night. (…) Then questions like where to get medical help, medical treatment, people don’t know it at all, especially, if they don’t have*

*insurance or personal identity documents.”* (A., male, 37 yrs., Latvian origin)

In Berlin, harm reduction services were accessible without identification documents. However, authorized insurance or being officially recognized as a refugee was required to access OAT and residential services, particularly for those unable to afford them independently. According to some Russian-speaking participants, one organization provided financial assistance for accessing drug services to individuals without official insurance or residence papers. Six participants indicated that they no longer wanted support because they distrusted certain institutions based on past experiences. One Islamic participant distrusted institutions that did not share his beliefs and suggested that a harm reduction service specifically tailored to Muslims would be a better fit for him.

#### Encounters with the criminal justice system and law enforcement

Five Russian-speaking, four North African and three West African PMWUD had been detained in Germany, because of theft (linked to rough living conditions and acquisitive crime), lack of a residence permit and amounting fines. Their experiences in German jail were surprisingly positive, probably because of the contrast with the rough living conditions outside prison. In prison, they had access to medical (OAT, tuberculosis and hepatitis C treatment), legal support (with IDs and residence permits) and social support. One Russian participant who had been incarcerated mentioned that she had learned German and had received help from some guards. However, three others regarded prison as a stepping stone towards substance use (“*You will leave prison as an expert in drugs”*) and criminal activities (*"Do this and we’ll give you some money…*”). Although one person indicated that he was supported well in prison, this was instantly discontinued upon release which resulted in homelessness, giving up HIV and OAT treatment and relapsing into heroin use after a period of controlled substitution.

*“I was staying there for nine months [in prison], and getting substitution therapy as well as medication for tuberculosis. (…) They cured me in nine months and that was it. Without any warning, they cut off my substitution therapy and stopped providing the HIV medication. All the help was cut off, I got kicked out and ended up on the street, and that was it. I started doing drugs again but didn’t have my HIV medication. I weighed 51 kilograms.”* (M., male, 42 yrs., Lithuanian background)

Many PMWUD had similar, mixed experiences with law enforcement in Berlin. They mentioned some positive encounters with police officers helping them out when they were in trouble, such as finding an OAT service, helping during a fight, and referring to services for asylum requests. A North African participant stated that German police were very respectful compared to French police. When checked for drug possession, drugs were mostly confiscated without any fine or arrest. However, two participants highlighted that drug confiscation provokes criminal behaviour as drug-dependent persons simply need drugs to survive.

### Paris

#### Characteristics of the sample

In Paris, 32 PMWUD were interviewed: sixteen originating from Georgia, ten from the non-Georgian Russian-speaking community (referred to as Russian-speaking PMWUD) and six from Somalia. All Georgian PMWUD (25–62 yrs.) only had Georgian nationality and had migrated between four months and 13 years ago. Ten Georgian PMWUD had no official ID, three indicated that they had an expired temporary residence document, and two had an asylum status. The non-Georgian Russian-speaking PMWUD (25–47 yrs.) originated from EU and non-EU member states (cf. Table 1) and had migrated 0.5–9 years ago, with one exception who migrated 16 years ago. The Latvian and Lithuanian PMWUD could reside in France legally. Among the other non-Georgian Russian-speaking participants, four had no official ID, and one had a temporary ID, impeding access to resources such as health insurance and employment. The Somali PMWUD (18–35 yrs.) had migrated to France 0.5–7 years ago. Four of them had a temporary residence permit, while the other two had no official ID which complicated their access to health care and social rights.

#### Substance use

All Georgian participants who identified as male reported using opioid substitution substances (Subutex®/buprenorphine or methadone), mainly provided by official services. Eight mentioned additional drug injection next to OAT and nine co-used cocaine and its derivatives. Most of the participants began using substances in their home country between the ages of 12 and 29. Six participants cited relief from stress, suppression of negative thoughts, and forgetting problems related to homelessness, psychological issues, and past trauma as their main reasons for substance use, which boosted their mood and energy. Due to their physical and mental dependence, they could not live without substances. Similar to Georgian PMWUD, non-Georgian Russian-speaking participants mostly used opiates by injection. Most used methadone in the context of OAT, some used additional heroin (n = 2), ‘street’ methadone (n = 1) or morphine (n = 1). Substance use was related to coping with psychological difficulties and physical cravings. The majority of Somali participants started using drugs in Europe. They primarily smoked crack cocaine and cannabis. They all used drugs on the streets, and their use was related to coping with the uncertainty, stress and boredom of being homeless.

*“I have been in Europe for the past nine years. I haven’t been able to secure a job, an ID nor a home and this made me fall into endless thinking. Then some friends suggested that I take drugs to help cope with the stress.”* (H., 28 yrs., male, Somali origin)

#### Social networks

Most Georgian participants had a supportive social network of caring (mostly Georgian) friends, though two mentioned that because their friends prioritized substance use over friendship, they could not be considered “real friends”. Family connections were rare due to migration and (stigma concerning) substance use. Three participants without a supportive network felt alone and depressed, with two expressing a need for social and emotional support. Similarly, four Russian-speaking and four Somali participants felt alone because they did not have “real friends” and lost family connections, although two Somali participants indicated that they had reliable friends.

#### Medical and mental health problems

The majority of Georgian PMWUD (n = 11) indicated that they had psychological problems such as depression, neuroses and psychoses, PTSD, and feeling stressed or anxious due to harmful living conditions and sleeping problems. Five Russian-speaking participants talked about severe psychological health issues such as depression and suicidal thoughts due to hardship in multiple life domains.

*“You know, when you can’t do anything in life, when you can’t find a job, or you have problems with your family, or with your relatives, then you have different thoughts in your head. You think about the fact that if you’re gone, it will be easier for your relatives.”* (U., male, 40 yrs., Latvian origin)

These psychological issues were often related to traumatic experiences such as war-related trauma, rape, and the loss of loved ones. Others indicated experiencing anxiety, stress, loneliness and sadness because of their unstable living conditions related to homelessness and/or substance use problems.

In both communities, several medical health problems such as dental issues, stomach problems, infections (often related to drug injection) as well as other complex medical problems such as hepatitis, HIV and heart, liver or lung diseases, were reported. Eight Georgian PMWUD were or had been hepatitis C-positive (five were treated) and one tested positive for tuberculosis and HIV (not yet treated). The Somali participants, who were relatively young, were less screened for communicable diseases than the other groups and did not report any medical needs. One person mentioned unstable mental health and depression as factors initiating substance use.

#### (Support) needs

Three Georgian PMWUD were homeless, and six stayed in a housing program, generally facilitated by an asylum status. Because most participants had some type of (temporary) shelter, their basic needs for a safe place to stay and access to resources for personal hygiene were mostly fulfilled. Participants whose basic needs were not fulfilled reported a significant detrimental impact on their well-being and substance use.

*“I don’t have a house. I can’t afford to lower the dosage and stop using the drug, due to the fact that I don’t have a home and I’m on the street and nothing changes. I am in the same situation as it was two, three years ago. (…) If I had a house, many things would change. I would give up medicine and drugs, I would take a more serious look at life. (…) I don’t have anything. They don’t even give me shelter. They don’t give me anything, not even a house, the procedure that goes on and that I get into this drug. I’m tired already. The end of such a life is death.”* (B., male, 48 yrs., Georgian background)

Some Georgian PMWUD indicated that they did not want to stay in France but needed an official ID to travel to the country they were meant to go to. Although most Georgian participants indicated that their basic needs were met to some extent, most needed additional financial support and help with finding a job to be financially independent. Six Georgian migrants needed help with regaining control over their lives by assisting with safer drug use (n = 2) or reduction of harmful drug use (n = 5).

Only one Russian-speaking participant lived on the streets, while the majority lived in housing programs, with family or in social housing. As such, all participants indicated that their basic needs for personal hygiene and a safe place to sleep were mostly fulfilled. They also had access to basic medical care, often provided by a harm reduction service. All Russian-speaking PMWUD indicated that they needed help with getting legal residence and other documents, such as insurance, disability benefits and work permits, in order to get access to various resources. To increase their quality of life, most indicated that the root causes of their psychological stress (such as unemployment, unstable life conditions, substance dependence and loneliness) should be addressed, rather than talking to a psychologist. One person expressed the need for addiction treatment, but most wanted only to reduce (harmful) substance use.

For Somali participants, housing was mentioned as a top priority and a prerequisite to stop using drugs and get a job. Three also expressed financial needs and the need for support in finding a job.

#### Harm reduction and other types of support

Most Georgian PMWUD received OAT, which they regarded as helpful, though regular side use of other substances was reported. While most Georgian and Russian-speaking participants indicated several unfulfilled needs, harm reduction services had added value in testing (and treatment) for HIV, hepatitis and tuberculosis. All Russian-speaking participants had been in contact with a harm reduction service, which was helpful for many of them. This could be related to the fact that a Russian-speaking counsellor was available at the service. Harm reduction services offered a place to seek help and provided support around basic, social and medical needs, in particular via the housing program, food supply and the DCR.

*“When you are left alone and you don’t know where to go, you go to the right, to the left, straight. When you are being supported not only in one aspect (…) that’s awesome, that’s a huge stimulation, a huge motivation.”* (A., female, 40 yrs., Latvian origin)

Similar needs were mentioned by Somali PMWUD, although they reported fewer contacts with drug services. At least three Somali migrants had not been tested for HIV, hepatitis or tuberculosis. Many PMWUD across the communities made use of ‘food tickets’ that allowed them to buy food at supermarkets.

#### Barriers to care and other types of support

Across the three communities, PMWUD experienced various barriers to social and health services, including language barriers, lack of accessible information, no insurance or other relevant documents, harsh living circumstances, enrolment procedures for drug treatment and substance dependence (e.g., by not being able to meet appointments) and (internalized) stigma. M. described how his undocumented status and the related stigma and fear of being deported kept him from care and support, making him feel help- and hopeless.

*“In general, I think, the main obstacle is my helplessness. My health and some physiological problems. I don’t really want to talk about it. Sometimes you’re just simply afraid, having these prejudices in your head, ‘You can’t do this. You can’t say that’.”* (M., male, 47 yrs., Tchetchen origin)

Regarding accessibility, a translation service and the provision of a map were suggested as solutions to help PMWUD with appointments. Substance dependence was mentioned as a barrier to learning the language and finding a job.

Russian-speaking participants pointed to their irregular status and not having health insurance as important barriers to medical care, income and housing. They mentioned a lack of continuity in social support services which impeded the development of helping relationships. Another Russian-speaking participant reported that he only got access to medical services when his medical issue was ‘severe’ enough. One participant mentioned that native French people were prioritized at the shelter. Additionally, external stigma evolving from the outer features of homelessness (*“old dirty clothes of vagrants”*) was mentioned as a barrier to support by a Somali participant.

#### Encounters with criminal justice and law enforcement

Most Georgian participants described their encounters with the police as stressful and fear-inducing which may be related to a language barrier and previous traumatic experiences with the Georgian police. Four Georgian participants explained that theft was an alternative means of self-sufficiency due to their limited financial resources. Similarly, three others were fined by the police for using public transport without paying. Other contacts with the police were related to substance use and being undocumented. In general, the police were rarely considered helpful. Furthermore, one participant indicated that in the case of a trial, it is difficult to be informed about it if one has no home address. Two participants had received a deportation order, while another feared deportation. Several Georgian and Russian-speaking participants reported stigma and discrimination by the police because of their migration background, drug use and past criminal offences.

*“You are not protected from anyone. Even if I want to complain, they tell me to leave. (…) The police may stop you for something, either you entered the subway without a ticket or something else and they insult you, looking at you as a commodity.”* (D., male, 55 yrs., Georgian origin)

Despite an agreement with the police that PWUD can carry a dose for their own use to the DCR, this was not always respected. Positive encounters with the law enforcement system included support by the police after encountering rape and not being arrested for drug possession even though carrying crack. Russian-speaking PMWUD generally had positive experiences with detention in France. They appreciated having access to a free lawyer and hepatitis treatment, being able to decrease substance use, as well as getting a meaningful job in prison. Somali participants reported no major problems with the police or judicial system, except for two who had been checked by the police for their documents, which resulted in one night of detention for one participant.

## Discussion

Based on 99 interviews conducted by community researchers in 14 different languages in four European metropolitan cities, we reached a diverse group of PMWUD. Several pressing needs regarding access to health care and social rights were identified. Support needs expressed by the participants were explored, as well as opportunities to break the cycle of precariousness that PMWUD often face. The limitations of the study are also addressed in this section, together with recommendations for further research.

### Access to health care for PMWUD

The UN Committee on Economic, Social and Cultural Rights states that healthcare facilities, services, and goods must be sufficiently available, easily accessible in terms of information and physical access, affordable for everyone, culturally sensitive, and of high quality. They also emphasize that discrimination based on any status must be strictly prohibited [49, 50].

While EU regulations mandate that asylum applicants receive necessary health care, including emergency care, essential treatment for illness, and necessary medical and other assistance for individuals with special needs [35], the provision of healthcare for undocumented migrants is governed by national policies. Therefore, access to emergency healthcare (including life-saving measures and treatments to prevent serious health damage, such as harm reduction services that provide OAT and prevent the spread of communicable diseases), primary healthcare (essential outpatient treatment for minor illnesses), and secondary healthcare (specialist and inpatient care) for undocumented migrants varies across different countries [51].

Health insurance is always mandatory to cover the costs of health care other than emergency care [28]. Nevertheless, several requirements for health insurance, such as having a home address and an income, may be hard to reach for PMWUD, implying that many of them do not have access to care other than what is considered emergency health care. While documented migrants are supposed to have the same access to healthcare and social services as native citizens, their access is often hindered by barriers such as personal, financial, legal (e.g., insurance requirements), cultural (e.g., stigma), and practical obstacles (e.g., language barriers, need for a home address, transportation) [1, 35, 52, 53].

Drug treatment is not explicitly mentioned in EU regulations and is often not prioritized in delivering healthcare to PMWUD. Legal access to harm reduction and drug services for undocumented PMWUD is dependent on individual countries and whether these countries consider substance dependence an essential ‘emergency’ health need [35]. For both documented and undocumented migrants, access to drug treatment services is limited due to a multitude of personal, financial, social, legal, cultural, geographical or practical barriers [6, 35]. This study confirmed that access to harm reduction services for PMWUD is dependent on the regulations, provision and accessibility of these services per country. Nevertheless, they have shown to be one of the few services that are able to overcome certain barriers to care for PMWUD.

### Access to social rights for PMWUD

Homelessness and poverty among persons with migration histories has become a matter of growing concern in many European countries, particularly with respect to asylum seekers and refugees, undocumented migrants and, increasingly, economic migrants from central and Eastern European countries [17]. Additionally, research indicates that substance use and dependence are strongly linked to homelessness [19]. This study confirms the vicious cycle of homelessness, poverty, substance use, criminal activities, irregularity and loneliness.

The precarious situations that PMWUD face in urban realities imply marginalization because they complicate and upset established norms and institutions [54]. In that regard, Misje (2021) points out that, among persons with migration histories who are homeless, the ‘precarious inclusion’ in public social welfare is often restricted to ensuring basic physical survival, albeit in an unpredictable and uncertain manner. This study confirms that legal access to care for PMWUD comes from a moral imperative to alleviate acute suffering, but insufficiently takes into account comprehensive social and human rights. Furthermore, it was confirmed that access to social rights often depends on multiple requirements, indicating that some individuals are considered more ‘deserving’ of human rights than others [55]. This implicit rationale, which tolerates distinctions in the values of individuals within the same context, seems to be accepted and reinforced by existing regulations within EU countries [56, 57]. Refugees from Ukraine, for example, receive more support in meeting certain human rights compared to other refugees, increasing their well-being, access to care, other types of support and related opportunities for recovery, societal inclusion, and personal and social development [58–60]. Additionally, migrants who formally reside and work in a country, and have sufficient financial means, have more access to health care and social welfare services than those who do not [57, 61].

Many PMWUD are struggling financially, physically and emotionally. Hence, from the current normative and neoliberal notion of citizenship, they face multiple barriers to so-called ‘productiveness’ (i.e., contributing financially to society through formal work), which has a major impact on ideas of deserving certain social rights [61, 62].

### (Support) needs

Research on migration and substance dependence frequently utilizes Maslow’s hierarchy of needs as a framework to evaluate the needs of individuals with a migration background and/or substance use problems [20, 63]. The results of this CBPR study show that many PMWUD in the EU are deprived of these needs due to barriers at multiple levels. According to Maslow’s model, basic physiological needs must be fulfilled before the higher-level needs of safety, belonging, esteem and self-actualization can be addressed [64]. While the need for food and basic hygiene was often addressed and mostly assessed as fulfilled by the participants, they also mentioned drug use as a necessity and basic need due to dependence. Additionally, they often lacked a stable home. Safety needs, as defined by Maslow (1943) include financial security, employment, personal security (i.e., protection from bodily harm), physical health, and well–being. Many PMWUD face challenges in fulfilling these needs, as they struggle with unemployment and contend with multiple physical and mental health issues that are often exacerbated by structural barriers to care [53]. Additionally, belonging needs are often unfulfilled for PMWUD, due to mechanisms related to migration and drug use, such as limited or unreliable social networks, stigma, discrimination and cultural differences [9, 22].

While lower-order needs such as overcoming drug dependence and homelessness are often emphasized as paramount among the needs of PMWUD, it is important to recognize that other needs may also be significant. As long as these urgent challenges persist, higher-level needs may remain hidden. PMWUD may be more sceptical toward the benefits of addressing higher-order needs if they conflict with their more pressing concerns [63]. However, this does not imply that higher-order needs are not at stake.

### Breaking the cycle

PMWUD experience difficulties in reaching both informal and formal support systems [9, 10, 65]. Without promoting access to care on a structural, social and personal level, PMWUD are inclined to remain trapped in a vicious cycle of precariousness.

Ensuring access to qualitative and humane healthcare for all PMWUD is not only a matter of human rights but could also be beneficial for the entire society in the long term. First, research indicates that preventive healthcare for persons with a migration history not only honours their human rights as adopted by the UN [50] but also reduces the public health burden [66]. Second, the abovementioned challenges that PMWUD face hinder their inclusion in society, preventing them from becoming accepted and valued citizens. By addressing the needs of PMWUD, such as issues with substance dependence, homelessness and legal aspects, by fostering a sense of hope and belonging (e.g., through community support and safe spaces), by reducing barriers to human rights, opportunities for positive change, improved well-being and recovery can emerge [25, 67–69]. Enhancing the availability and accessibility of health and social services for all PMWUD protects human rights, advances public health, and supports social inclusion [5].

This study highlights that harm reduction and other specialized services can significantly contribute to the health and well-being of PMWUD. They provide essential material, social, and emotional resources, offering consistent support in the face of unstable health, living, and social conditions [70]. As harm reduction services are at the forefront and often among the few providers of support for PMWUD, investment in these services is essential [14].

Good practices to overcome some of the barriers to care PMWUD face, such as outreach services and representation of PMWUD in harm reduction services, were identified. The representation and participation of PMWUD in the organization of (mental) health provision could further enhance its accessibility and efficacy for this population [71].

### Limitations and recommendations for future research

Some limitations of this research need to be acknowledged. The first set of limitations relates to the CBPR approach. CBPR is designed to involve and collaborate with individuals whose life experiences are the focus of the study, both in planning and conducting the research process [72]. However, this was not fully possible. First, the research proposal was written before most of the community researchers were identified, limiting their input in the first phases of the research process. Second, there was a language barrier between the academic and community researchers. Third, strictly speaking, the community researchers in this study should have been all PMWUD. However, this study highlighted the precarious situations many of these individuals face. Focused on everyday survival, there is little room for other engagements. Hence, local researchers, as do many researchers conducting participatory research, faced challenges in recruiting PMWUD to strengthen the research team [39]. Ultimately, the community researchers had lived experiences as migrants, persons who use(d) drugs or as PMWUD. Future research could enhance participation by involving more PMWUD throughout the entire process, from start to finish, conducting the research at the community researchers’ pace and tailoring it to their specific needs.

For pragmatic and practical reasons, we chose to focus on three communities in each city. These communities, however, were often broadly categorized and comprised a very heterogeneous group of people with many other intersecting aspects of identity. Additionally, the small sample sizes of the communities (which may not be regarded as a ‘community’ per se by the participants) indicate that the results are not generalizable to these communities.

Finally, even though we aimed to include gender minorities in this research, the number of participants other than cisgender men was very limited, allowing for only a few gender-specific statements. Future research should focus on the specific experiences and needs of gender-minority PMWUD. Moreover, exploring good practices for supporting PMWUD is essential. Additionally, examining how Ukrainian refugees are treated under EU and local legislation, and how these laws provide access to personal and social resources benefiting both Ukrainian refugees and societies in the long term, may inform the development of future policies to ensure the best possible reception of refugees. Longitudinal research may uncover how the decisions made by practitioners and policymakers today impact the lives of PMWUD in the future.

## Data Availability

The data are not publicly available due to ethical and privacy reasons.

## Abbreviations

PMWUD: Persons with a first-generation migration background who use drugs
CBPR: Community-based participatory research
OAT: Opioid agonist therapy
DCR: Drug consumption room

## Footnotes

1 The SEMID-EU project was funded by the European Union’s Justice Programme — Drugs Policy Initiatives (Grant agreement number 101045837). It was coordinated by Mainline, and exists of four work packages including 1) Assessing the current situation through a systematic review of the literature and database analyses (by IS Global), 2) Developing an inclusive understanding of local needs and responses through a CBPR study (by Ghent University and the local partners), 3) Crafting policy recommendations, toolkits and practical guidelines through factsheets and policy papers (by Correlation Network), and 4) Implementing these insights in daily practice through local capacity-building workshops (by local partners). The local partners were Mainline (Amsterdam), Positive Voice (Athens), Fixpunkt e.V. (Berlin) and Gaïa (Paris). All outcomes of the SEMID-EU project can be found here: https://mainline.nl/en/projects/migrants-drug-use/.
